# Intestinal flora and bile acid interactions impact the progression of diabetic kidney disease

**DOI:** 10.3389/fendo.2024.1441415

**Published:** 2024-09-20

**Authors:** Jia Xu, Nan Wang, Li Yang, Jing Zhong, Ming Chen

**Affiliations:** Department of Nephrology, Affiliated Hospital of Chengdu University of Traditional Chinese Medicine, Chengdu, Sichuan, China

**Keywords:** diabetic kidney disease, intestinal flora, bile acids, farnesoid X receptor, G protein-coupled bile acid receptor 1, exosomes

## Abstract

In recent years, with the rapid development of omics technologies, researchers have shown that interactions between the intestinal flora and bile acids are closely related to the progression of diabetic kidney disease (DKD). By regulating bile acid metabolism and receptor expression, the intestinal flora affects host metabolism, impacts the immune system, and exacerbates kidney injury in DKD patients. To explore interactions among the gut flora, bile acids and DKD, as well as the related mechanisms, in depth, in this paper, we review the existing literature on correlations among the gut flora, bile acids and DKD. This review also summarizes the efficacy of bile acids and their receptors as well as traditional Chinese medicines in the treatment of DKD and highlights the unique advantages of bile acid receptors in DKD treatment. This paper is expected to reveal a new and important potential strategy for the clinical treatment of DKD.

## 1 Introduction

**Figure 1:**
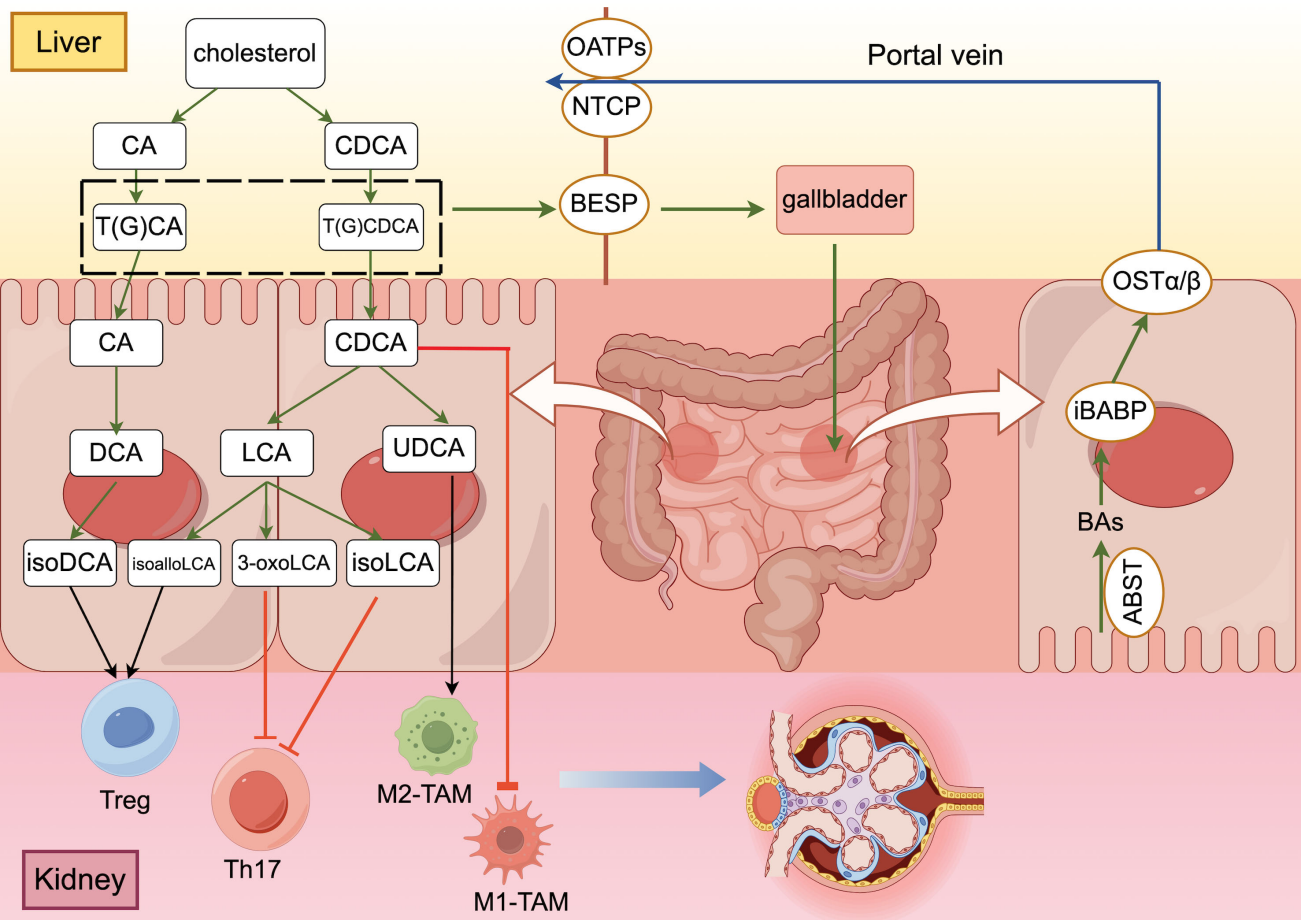
The “gut-liver-kidney axis” in patients with DKD ((generated by Figdraw 2.0). Primary conjugated bile acids are synthesized into secondary bile acids by deconjugation, dehydroxylation, oxidation and differential isomerization, and the intestinal flora participates in this process. Bile acids that are synthesized and stored in the liver are transported to the intestine via transporter proteins and transferred to hepatocytes with the help of other transporter proteins, completing the “enterohepatic cycle” in the human body. After bile acids perform their functions in the intestine, they promote the polarization of Treg cells and M2 macrophages and inhibit the differentiation of Th17 cells and the polarization of M1 macrophages through different signaling pathways to maintain the homeostatic environment of cellular immunity.

With the increasing prevalence of diabetes mellitus worldwide, DKD, which is a serious complication of diabetes, has become one of the top ten causes of death around the world. The global prevalence of diabetes mellitus was reported to be 463 million in 2019, and it is expected to increase by 25% by 2030 and 51% by 2045. Approximately 20–30% of all diabetic patients around the world develop DKD, and approximately 50% of these patients progress to end-stage renal disease (ESRD) (1, 2). The pathogenesis of DKD is complex. The inflammatory response, renal haemodynamic disorders and the imbalance of immune homeostasis can cause glomerular histiocyte and endothelial cell injury, glomerular basement membrane thickening, and glomerulosclerosis, ultimately leading to renal fibrosis and exacerbating renal injury. Glycolipid metabolism disorders are also important causes of DKD. High glucose levels increase AGE production, and the interaction of AGEs with their receptors causes oxidative stress, which increases the production and release of inflammatory factors and causes an inflammatory response. Hyperglycaemia interferes with cholesterol synthesis and bile acid metabolism, exacerbating renal lipid accumulation; this renal lipid accumulation, in turn, causes Extracellular matrix (ECM) accumulation, further worsening glomerulosclerosis and renal fibrosis (3). Increasing evidence suggests that the gut flora and bile acids synergistically regulate host energy metabolism and immune responses via the “gut-liver-kidney axis”, thereby accelerating the progression of DKD (**Figure 1**). However, the exact mechanisms involved remain unclear. Therefore, understanding interactions between the intestinal flora and bile acids and elucidating the pathological mechanisms involved in DKD progression are important for the treatment and prevention of DKD. In this paper, we aim to provide a detailed review of this issue, focusing on the effects of bile acids and receptors on DKD that are mediated by different metabolic and inflammatory pathways, interventions and mechanisms that regulate these pathogenic factors, and the use of traditional Chinese medicine to slow DKD progression. Our goal is to provide a theoretical basis for DKD treatment and intervention and offer new ideas to improve the clinical outcomes of DKD patients.

## 2 Disturbance of the intestinal flora disrupts bile acid homeostasis in DKD patients

### 2.1 DKD is characterized by disruption of intestinal flora homeostasis

Approximately 500 different bacterial species are present in the intestinal tract of healthy individuals, with a predominance of anaerobic bacteria, including Firmicutes, Bacteroidetes, Actinobacteria, Proteobacteria, Fusobacteria and Verrucomicrococcus; among these phyla, Firmicutes and Bacteroidetes account for 90% of the bacteria in the intestinal tract. The intestinal flora plays an important role in regulating human metabolism and maintaining immune homeostasis, and it is involved in the progression of many metabolism-related diseases (4). Previous studies have shown that DKD causes changes in the structure of the intestinal flora. Compared with healthy individuals, DKD patients exhibit significant differences in the structure of their microbiota; these differences are characterized by notable changes in α and β diversity (5–7), and exacerbate the disruption of gut microbial homeostasis. In recent years, the intestinal flora has been shown to be involved in the development of DKD. Li et al. (8) conducted 16S rDNA gene sequencing on the intestinal flora of DKD model mice and reported a decrease in the abundance of Firmicutes, such as Lachnospiraceae, Lactococcus, Fusobacterium, and Lactobacillus, whereas the abundance of Bacillus increased, exhibiting a positive correlation with albuminuria levels. Tao et al. (9) r reported that the abundance of Prevotella and Bifidobacterium was lower in DKD patients than in healthy individuals. Wang et al. (6) reported a reduction in the abundance of Roseburia, which is an Actinobacteria, and Akkermansia muciniphila, which is a member of the phylum Verrucomicrococcus, in the intestinal tracts of DKD patients. Moreover, other studies revealed increased abundances of Klebsiella and *Escherichia coli*, as well as the phylum Aspergillus (10–14). All the aforementioned studies indicate that DKD is closely associated with disordered gut microbial ecology and that DKD patients have a decreased diversity of gut flora compared with healthy individuals, as evidenced by a decrease in the abundance of beneficial bacteria and an increase in the abundance of pathogenic bacteria (**Table 1**).

**Table 1:** Evidence from studies of changes in intestinal flora in patients with DKD and effects on bile acids.

Phylum	Changes of genus level in DKD	Influence on BAS	Changes of BAs DKD	Ref.
Firmicutes	Lachnospiraceae↓	The number of bai operators required for 7α- dehydroxylation is reduced	LCA↓	(8, 21)
	Lactococcus↓			
	Fusobacterium↓			
	Lactobacillus↓			
	Bacillus↑			
Bacteroides	Bifidobacterium↓	Decreased BSH activity	conjugated BAs(TCA/GCA/TCDCA/GCDCA)↑ Unconjugated BAs(CA/CDCA)↓	(9, 19)
Actinobacteria	Roseburia↓	Decreased HSDH activity and the isomerization of bile acids 3α-, 7α- and 12α- was inhibited from α- to β- direction	UDCA↓	(6, 19)
Verrucomicrobia	Akkermansia muciniphila↓			(6)
Proteobacteria	Klebsiella↑	Decreased 7α/β-dehydrogenase activity and inhibit dehydroxylation	DCA↑	(12, 20)
	Escherichia coli↑	ASBT transporter function is inhibited	Obstruction of enterohepatic circulation caused by cholestasis	(10)

↓: decreased; ↑: increased.

### 2.2 Imbalances in intestinal flora homeostasis can lead to disturbances in bile acid metabolism in DKD patients

Bile acid (BA) is an amphiphilic substance that is synthesized from cholesterol in the liver and plays crucial roles in glycolipid metabolism and immune regulation (14). In the human body, BA produces cholic acid (CA) and cyanodeoxycholic acid (CDCA) through classical pathways, while CDCA is also produced through alternative pathways (15, 16). In rodents, generated CDCA is immediately converted into rhombocholic acid (αMCA and βMCA) (16). Unlike that in humans and mice, primary BAs in pigs are dominated by hyocholic acid (HCA). The resultant primary free BA combines with glycine and taurine at a ratio of 3:1 to form binding BAs (17), such as glycincholic acid (GCA), taurocholic acid (TCA), glycochenodeoxycholic acid (GCDCA), and taurochenodeoxycholic acid (TCDCA). An increasing number of studies have shown that there is a significant correlation between dysregulation of the serum bile acid profile in DKD patients and disruption of the intestinal flora and that the interaction between the two affects the development of DKD. Xiao et al. (18) analyzed the serum bile acid components of 184 patients with biopsy-confirmed DKD and reported that low bile acid levels were significantly correlated with increased proteinuria and decreased eGFR levels. Li et al. (19) observed an increase in the proportion of bound bile acids (TCA, GCA, TCDCA, and GCDCA) in the serum of ESRD patients, while the content of free bile acids (CA, CDCA and UDCA) decreased. Mantovani et al. (20) reported that the level of deoxycholic acid (DCA) was increased in patients with glucose metabolism disorders, and Li et al. (21) reported that the level of lithocholic acid (LCA) was decreased in rats fed a high-fat diet (**Table 1**). As mentioned in section 1.1, intestinal flora homeostasis in DKD patients is disrupted, resulting in a decrease in the level of enzymes involved in bile acid biotransformation; in turn, this decrease affects secondary bile acid synthesis, bile acid cyclic metabolism and bile acid receptor expression.

#### 2.2.1 Disruption of the intestinal flora affects secondary bile acid synthesis

Under the action of the intestinal flora, the primary BA in the physiological state is first uncoupled by bile saline hydrolysase (BSH) (22) and then forms secondary BA after 7α-dehydroxylation; this product is transformed into DCA and LCA in humans, into mouse deoxycholic acid (MDCA) in mice, and pigs generate hyodeoxycholic acid (HDCA) (23). In the human body, certain bacteria produce hydroxysteroid dehydrogenase (HSDH) to isomerize CDCA to UDCA, which can also convert LCA to iso-stone cholic acid and oxystone cholic acid. In the mouse body, β-MCA is hetero-isomerized to form ω-MCA at the C-6 position (25–27).

Studies have shown that BSH is widely active in gram-positive bacteria such as Bifidobacterium, Lactobacillus, Enterococcus, and Brucella and can also be detected in gram-negative bacteria such as Bacteroidetes (24–27). The bai operon is a key structure that regulates gene expression, and scientists have found that the enzyme it encodes plays an important role in the dehydroxylation of cholic acid. Researchers have found that bai operators are required for 7α-dehydroxylation in Firmicutes and Clostridium, and a 7α/β-dehydrogenase capable of converting CA and CDCA into DCA and LCA after dehydroxylation has been found in Proteobacteria and Erysipelothrix (26, 28, 29). HSDH in Actinomycetes and Proteobacteria can catalyze the isomerization of the bile acid 3α-, 7α- and 12α-hydroxyl groups from the α-direction to the β-direction, thereby increasing the hydrophilicity of bile acids (25). Researchers have also shown that Egeria tarda and Clostridium perfringens are involved in bile acid oxidation and differential isomerization (26, 27). However, due to the decrease in the abundance of Bifidobacteria, Firmicutes and Actinobacteria in the intestinal tract of DKD patients, the activities of enzymes such as BSH and HSDH are reduced, and the dissociation and isomerization of conjugated bile acids are blocked, resulting in increased cholestasis and the exudation of inflammatory substances, thus aggravating the systemic inflammatory response. The above studies indicated that intestinal flora homeostasis in DKD patients affects the activity of bile acid-metabolizing enzymes, leading to disruption of secondary bile acid synthesis.

#### 2.2.2 Disruptions in the intestinal flora affect bile acid circulation and metabolism

Synthesized BA is metabolized in the human body via interactions between the liver and the intestine and plays a crucial role in the absorption of nutrients and energy metabolism throughout the body (29). BA bound to the liver is secreted into the bile duct through the bile salt output pump (BSEP) and stored in the gallbladder. After eating, the gallbladder is stimulated to contract and release BA into the intestinal lumen to promote the emulsification and absorption of lipids and fat-soluble vitamins (22). Then, the apical membrane sodium-dependent BA transporter (ASBT) is reabsorbed into intestinal epithelial cells, the intracellular ileal BA binding protein (iBABP) is transported to the basolateral side of intestinal epithelial cells, and the organic solute transporter (OSTα/β) transfers BA through the basolateral membrane to the portal vein (15). Subsequently, sodium-taurocholic acid cotransporter peptides (NTCP) and organic anion transporter peptides (OATPs) are involved in transporting BA from the blood to liver cells, and the reabsorbed BA is again secreted into the bile duct with the newly synthesized BA to complete a complete enterohepatic cycle (30) (**Figure 1**). In the human body, the enterohepatic cycle occurs approximately 4-12 times a day, and only 5% of BA is excreted in the stool (31).

Intestinal flora disruption results in abnormal bile acid metabolism by affecting the expression of bile acid transporters (32). *Escherichia coli* is an opportunistic pathogen, and the abundance of *E. coli* is significantly increased in the intestines of DKD patients. Annaba et al. (33) reported that *E. coli* inhibit the transport function of ASBT by inducing the secretion of proinflammatory factors and reducing tyrosine phosphorylation. In an animal study, Out et al. (34) reported that intestinal flora disorders inhibited ASBT expression by activating Gata4, thus disrupting bile acid reabsorption and cholestasis in the intestine, worsening intestinal damage and exacerbating inflammatory responses.

#### 2.2.3 Disruption of the intestinal flora affects bile acid receptor expression

Farnesoid X receptor (FXR) and Takeda-G-protein-receptor-5 (TGR5) are important bile acid receptors that play important roles in host metabolism and immune regulation. According to human and animal model studies, FXR is a member of the nuclear receptor superfamily and is widely expressed in the liver, kidney and small intestine (36–40), whereas TGR5 belongs to the membrane receptor family and is highly expressed in the liver, gallbladder, intestine and kidney (41–43). A recent study revealed that FXR-/TGR5- db/db mice presented elevated levels of creatinine, uric acid, and proteinuria (41), suggesting that limited bile acid receptor expression in DKD patients may exacerbate kidney injury.

As the endogenous ligand of FXR/TGR5, bile acids have different excitatory or inhibitory effects on this receptor. Disruption of the intestinal flora further affects FXR/TGR5 expression by altering bile acid homeostasis. In the liver, the ability of bile acids to activate FXR is as follows: CDCA>DCA>LCA>CA (42, 43); however, UDCA, Tα-MCA, and Tβ-MCA are considered natural antagonists of FXR (44–46). The secondary bile acids LCA and DCA have the strongest excitatory effects on TGR5, followed by CA and CDCA (46, 47). As mentioned in section 1.2.1, intestinal flora homeostasis in DKD patients caused a decrease in the abundance of Bifidobacterium and Lactobacillus, which produce BSH, leading to an obstruction of the dissociation of Tβ-MCA in mice and an increase in the level of Tβ-MCA *in vivo*, thus increasing the antagonism of FXR. In addition, the production of 7α-dehydrogenase by Bacteroides is decreased in the guts of DKD patients, resulting in a corresponding decrease in LCA conversion; this decrease suppresses the excitatory activity of TGR5. Furthermore, drugs affect the distribution of the intestinal flora, which in turn affects bile acid receptors. Metformin is currently the most common hypoglycaemic drug, and metformin can affect the structure of the intestinal flora and inhibit the activity of FXR in the intestine. When T2D patients take metformin, the activity of BSH produced by *Bacteroides fragilis* in the intestine decreases, resulting in increased levels of glycoursodeoxycholic acid (GUDCA) and tauroursodeoxycholic acid (TUDCA). GUDCA and TUDCA are considered effective FXR antagonists that ameliorate insulin resistance by inhibiting FXR activity in the intestine (45).

## 3 Disruption of bile acid metabolism can accelerate the progression of DKD

Studies have shown that bile acid metabolism disorders can result in energy metabolism and cellular immune dysfunction in DKD patients by interfering with glucose and lipid metabolism and immune cell function, including macrophage polarization and Th17 and Treg cell differentiation; these effects ultimately accelerate the progression of DKD.

### 3.1 Bile acid metabolism disorders affect energy metabolism in patients with DKD

Studies have shown that changes in the bile acid spectrum in DKD patients can lead to abnormal levels of glucose and lipid metabolism indicators. A study examining the correlation between changes in total bile acid (TBA) and glucose metabolism revealed that increased TBA levels are positively correlated with the HOMA-IR index (48). In another study, Haeusler et al. (49) investigated plasma BA levels and insulin sensitivity in 35 T2DM patients and reported that an increased ratio of 12α-hydroxyl/non-12α-hydroxyl BA is accompanied by decreased insulin sensitivity and increased triglyceride levels. Further tests revealed that an increase in 12α-hydroxy-BA levels exacerbates insulin resistance in T2DM patients; thus, we believe that a change in 12α-hydroxy-BA levels is the main factor that impacts DKD-related glucose metabolism indices. Studies have shown that bile acids can increase glucose uptake by tissues and help maintain systemic blood glucose homeostasis by activating FXR/TGR5 (50, 51). In an animal study, Wang et al. (52) reported that triglycerides and cholesterol accumulate in FXR-knockout mice and that glomerulosclerosis and albuminuria are exacerbated in these mice. After the administration of the FXR agonist INT-747 to diabetic mice, proteinuria is significantly reduced, and glomerulosclerosis and renal tubulointerstitial fibrosis are significantly alleviated. Thus, FXR activation can reverse kidney injury and delay DKD progression. In addition, researchers have reported that mice with FXR deficiency are more likely to develop kidney damage in the context of high glucose levels. According to section 1.2, the bile acid metabolism of DKD patients is disrupted, which suppresses FXR/TGR5 activation and prevents FXR/TGR5 from regulating energy metabolism.

### 3.2 Bile acid metabolism disorders affect immune function in DKD patients

#### 3.2.1 Bile acid metabolism disorders affect macrophage polarization

Macrophages are innate immune cells that can phagocytose pathogens, present antigens and secrete inflammatory substances. There is a significant correlation between DKD and macrophages. Compared with those in healthy individuals, many macrophages are enriched in the glomerulus and renal interstitium of DKD patients, and the degree of infiltration is positively correlated with renal damage indices, such as creatinine levels and proteinuria (53). These results suggest that macrophages are involved in inducing inflammation in the early stages of DKD and mediating kidney injury. Additionally, changes in the serum bile acid concentration in DKD patients can influence the polarization of macrophages, thus affecting the immune balance within the body.

Wang et al. (54) induced M1 macrophage infiltration after DCA supplementation in mice with inflammation *in vivo*. The mechanism may include DCA targeting Toll-like receptor 2 (TLR2) through the M2-mAchR/Src pathway and activation of the downstream ERK/JNK/NF-κB signaling pathway following TLR2 phosphorylation. This activation induces the polarization of macrophages, resulting in the mass recruitment of M1 macrophages; the release of TNF-α, IL-6, IL-8, IL-12 and other proinflammatory factors; and the exacerbation of inflammatory damage in DKD patients. Cao et al. (55) reported that increasing the CDCA level in mice could stimulate FXR activity, thereby inhibiting M1 macrophage polarization and IL-6 expression and exerting anti-inflammatory effects. Other studies have confirmed that UDCA inhibits intestinal inflammation and alleviates inflammation by inducing M2 macrophage polarization (56). After UDCA supplementation in ob/ob mice, Chen et al. (57) reported significantly reduced expression of M1 markers, such as NF-κB, IL-6 and TNF-α; increased expression of M2 markers, such as CD206, CD204 and CD163; and a decrease in the M1/M2 ratio; these phenomena alleviated the kidney injury caused by inflammation. As noted in section 1.2, DKD patients have increased DCA levels and decreased CDCA and UDCA levels, which can promote the polarization of M1 macrophages and inhibit the polarization of M2 macrophages; these effects induce the production of many proinflammatory factors and exacerbate the inflammatory response in DKD patients.

#### 3.2.2 Bile acid metabolism disruption affects the differentiation of Treg and Th17 cells

BA is also involved in the regulation of adaptive immunity and the differentiation of T cells, including Treg cells and Th17 cells. Treg cells, which are a subset of CD4^+^ T cells (58), play crucial roles in immune regulation by suppressing immune responses. Foxp3 is an important transcription factor in the development of Treg cells. Treg cells expressing Foxp3 can secrete TGF-β and other anti-inflammatory factors, which play important roles in maintaining immune homeostasis (59, 60). Th17 cells differentiate from CD4^+^ T cells after inducing RORγt expression and activating IL-17A in response to high concentrations of TGF-β. Th17 cells mainly secrete proinflammatory factors such as IL-17, IL-21, and IL-22, and these cytokines recruit neutrophils to the site of inflammation and exacerbate inflammation. Under physiological conditions, the relative balance between Treg cells and Th17 cells helps maintain immune homeostasis within the body (61).

**Figure 2:**
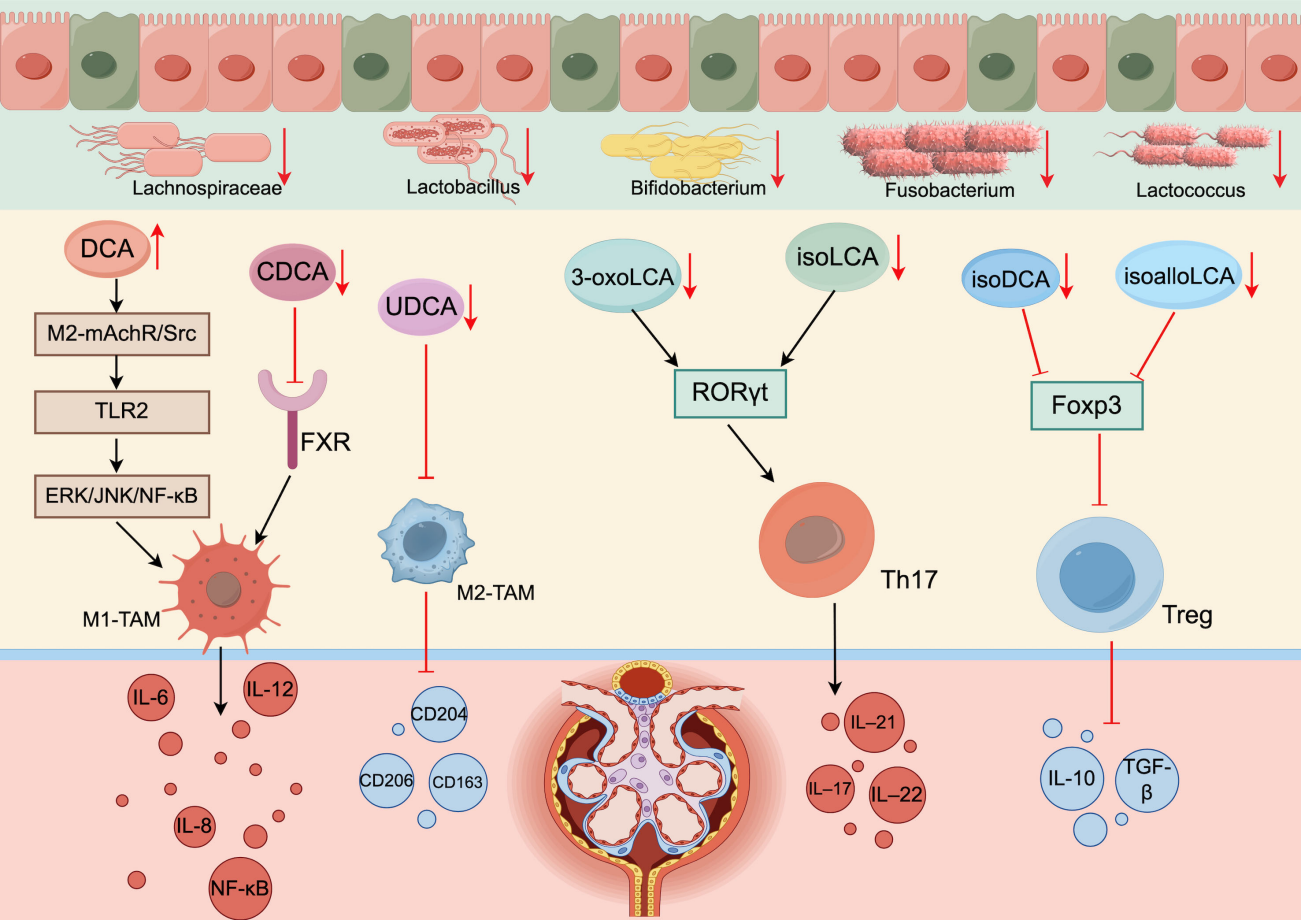
Disturbances in bile acid metabolism disrupt immune cell homeostasis (generated by Figdraw 2.0). When the intestinal flora of DKD patients is disrupted, the number of bacteria involved in bile acid synthesis and enterohepatic circulation is reduced, which affects the activity of various enzymes and reduces secondary bile acid synthesis. Bile acid acts as the endogenous ligand of FXR/TGR5; thus, reduced bile acid synthesis inhibits the activation of FXR/TGR5. In addition, an imbalance in the intestinal flora disrupts bile acid metabolism by inhibiting ABST expression.

Many recent studies have reported that an imbalance in bile acid homeostasis in DKD patients leads to a decrease in the proportion of Treg cells and an increase in the proportion of Th17 cells, which exacerbates inflammation and further affects the progression of DKD (62). Song et al. (63) reported that changes in the bile acid pool affect the expression of the transcription factors RORγt and Foxp3 and further affect the regulation of immune cells, resulting in increased colon inflammation. Researchers further investigated and reported that lithocholic acid derivatives affect Th17 and Treg cell differentiation in mice. 3-oxoLCA and isoLCA inhibit RORγt transcriptional activity and thus inhibit Th17 differentiation, whereas isoalloLCA and isoDCA promote Treg cell differentiation by increasing Foxp3 expression (64–66). As stated in section 1.2.1, a decrease in the number of bacteria that produce HSDH in the intestinal tract of DKD patients prevents the isomerization of stone cholic acid and reduces the production of stone cholic acid derivatives, thus suppressing Treg differentiation and the inhibition of Th17 differentiation, disrupting the immune balance of Treg/Th17 cells, and exacerbating the disorder of the immune environment in DKD patients (**Figure 2**).

## 4 Delaying the progression of DKD through the “gut-liver-kidney axis”

### 4.1 Regulating the homeostatic pathway of intestinal flora delays DKD

Through the exploration of the relationship between disease and intestinal flora, it has been found that the structure and composition of intestinal flora influence the development of metabolic diseases, and intestinal flora is both a pathogenic factor and a therapeutic tool. A recent clinical trial found that the probiotic Bifidobacterium longum NCC3001 increased levels of GUDCA and free fatty acids, as well as reduced depression scores and improved anxiety responses in patients. This suggests that there is a correlation between human subjective stress and intestinal flora (67). In a recent study that sequenced the 16S rRNA gene in stool samples from 56 Tibetan Buddhist monks and neighboring residents, researchers found that Prevotella and Bacteroides were significantly enriched in the meditation group, at 42.35% and 6.21%, respectively, compared with 29.15% and 4.07% in the control group. They also found significant reductions in clinical risk factors in the meditation group, including total cholesterol and apolipoprotein B; a separate study also found that LDL levels were significantly lower in the meditation group, and inflammatory genes including NF-KB-2 and IL1-B were 0.3 and 0.2 times higher, respectively, than in the control group (68). In addition to this, most of the research on the treatment of DKD by intestinal flora has focused on probiotics, prebiotics and fecal microbiota transplantation (FMT), which can treat and prevent DKD by regulating glucose metabolism and protecting the intestinal barrier.

#### 4.1.1 Probiotics and prebiotics

Probiotics and prebiotics have been considered effective treatments for DKD in recent years. Probiotics, which are intestinal microorganisms, can regulate the distribution of the intestinal flora and ameliorate intestinal flora disorders. Prebiotics are nondigestible food components that are usually degraded by gut microbes to provide nutrients to the gut but cannot be absorbed by the gut; these components restore gut health by stimulating the activity of beneficial bacteria in the gut (69). Both probiotics and prebiotics are significantly decreased in DKD patients, and supplementation with both can improve glucose metabolism, restore intestinal mucosal function, and reverse renal tissue structural damage in DKD patients.

*Lactobacillus royale* LR6 was found to increase the number of cuprocytes and protect the integrity of the intestinal mucus and lamina propria layers (70). Taverniti et al. (71) reported that *Bifidobacterium bifidum*, *Lactobacillus suis*, and *Lactobacillus casei* decrease zonulin expression and promote intestinal barrier repair. Second, probiotics have been shown to have an ameliorative effect on glucose metabolism markers, including FBG, HbA1c and HOMA-IR, in diabetic patients (72). After researchers treated diabetic mice with four strains of Lactobacillus probiotics for 8 weeks, the mice presented decreases in both FPG and HOMA-IR levels and significant increases in both GLP-1 and GLUT-2 expression (73). In addition, researchers orally administered two probiotics, *Bifidobacterium bifidum* BL21 and *Lactobacillus lactis* LRa05, to T2DM model mice, and these treatments reduced the expression of IL-17, IL-6, and endotoxin and ameliorated oxidative stress and inflammation in these mice (74). The mechanism involves blocking the TLR4/MyD88 signaling pathway by inhibiting the specific binding of LPS to TLR4, which ultimately prevents the expression of NF-κB signaling pathway components and reduces the production of proinflammatory factors, such as IL-2, IL-6, and TNF-α.

#### 4.1.2 FMT

FMT involves the transplantation of feces from a healthy person into a patient to remodel the patient’s intestinal microbial environment with the healthy flora of the donor. With in-depth research on FMT, an increasing number of studies have concluded that FMT is an effective treatment for dysbiosis, and in 2013, FMT was approved for the treatment of *Clostridium difficile* infection in the United States; this advanced the prospects of FMT to new heights (75).

Several studies have shown a significant increase in the diversity of the intestinal flora after FMT, and the levels of Ackermannia mucinophilic, Mycobacterium anisopliae and Mycobacterium-like organisms, which are important beneficial bacteria in the intestinal tract and are thought to inhibit intestinal inflammation, were increased after FMT. Zhang et al. (76) reported that the levels of fasting blood glucose, triglycerides and low-density lipoprotein cholesterol were reduced in db/db mice treated with FMT, suggesting that FMT has a positive regulatory effect on glucose and lipid metabolism in DKD patients. In a Phase 2 clinical trial predicting obesity and FMT-related traits, researchers found a significant increase in bacterial abundance in recipients after FMT and observed a significant improvement in HOMA2-IR at week 6 (77). In addition, The researchers studied FMT in 11 kidney transplant recipients and found that FMT treatment reduced drug resistance in the body by inhibiting the same strain (78). The results indicated that FMT was effective in improving intestinal bacterial richness and glucose metabolism. Zhao et al. (79) also reported that FMT reduced the level of LPS in the colons of mice, thereby inhibiting the activation and expression of NF-κB and reducing the production of proinflammatory factors such as TNF-α, IL-1β, IL-6, and IL-17. In addition, they reported that FMT improved the distribution of ZO-1 and tight junction proteins and restored intestinal barrier function in colitis model mice, suggesting that FMT plays an important role in regulating inflammatory pathways and alleviating inflammatory symptoms.

#### 4.1.3 Exosomes

Exosomes, which are vesicular vesicles secreted by a variety of cells containing various nucleic acids, proteins, lipids, amino acids, and metabolites, are considered to be important mediators of intercellular communication because they can participate in the exchange of information and substances between different cells (80, 81). Researchers first observed that exosomes secreted by intestinal bacteria could transport biomolecules into host cells after introducing Escherichia coli into mice (82). Fizanne et al. (83) found that feces-derived extracellular vesicles were able to reduce the expression of tight junction proteins in the intestines of wild mice, increase intestinal permeability which exacerbated intestinal inflammation. The above studies demonstrated the interaction between exosomes and gut microorganisms, which together affect the development of diseases. Subsequently, Teng et al. (84) found that ginger exosomes-like nanoparticles could induce Lactobacillus rhamnosus to activate the AHR signaling pathway and thus mediate the production of IL-22, which could ameliorate intestinal inflammation and restore the intestinal barrier function. In addition, Zhu et al. (85) found that plant-derived exosome-like nanoparticles increased Lactobacillus reuteri levels and promoted indole derivative-activated reprogramming of CD4^+^ T cells into double-positive CD4^+^CD8^+^ T cells, reducing the levels of pro-inflammatory causative cells. Yan et al. (86) also found that combining mesenchymal stem cells-derived exosomes (hUC-Exos and hFP-Exos) transplanted into colitis mice and found that hUC-Exos and hFP-Exos ameliorated intestinal inflammation by regulating the proportional balance between Th17 and Treg cells.

### 4.2 Reversing DKD bile acid metabolism disorder can delay the progression of DKD

**Figure 3:**
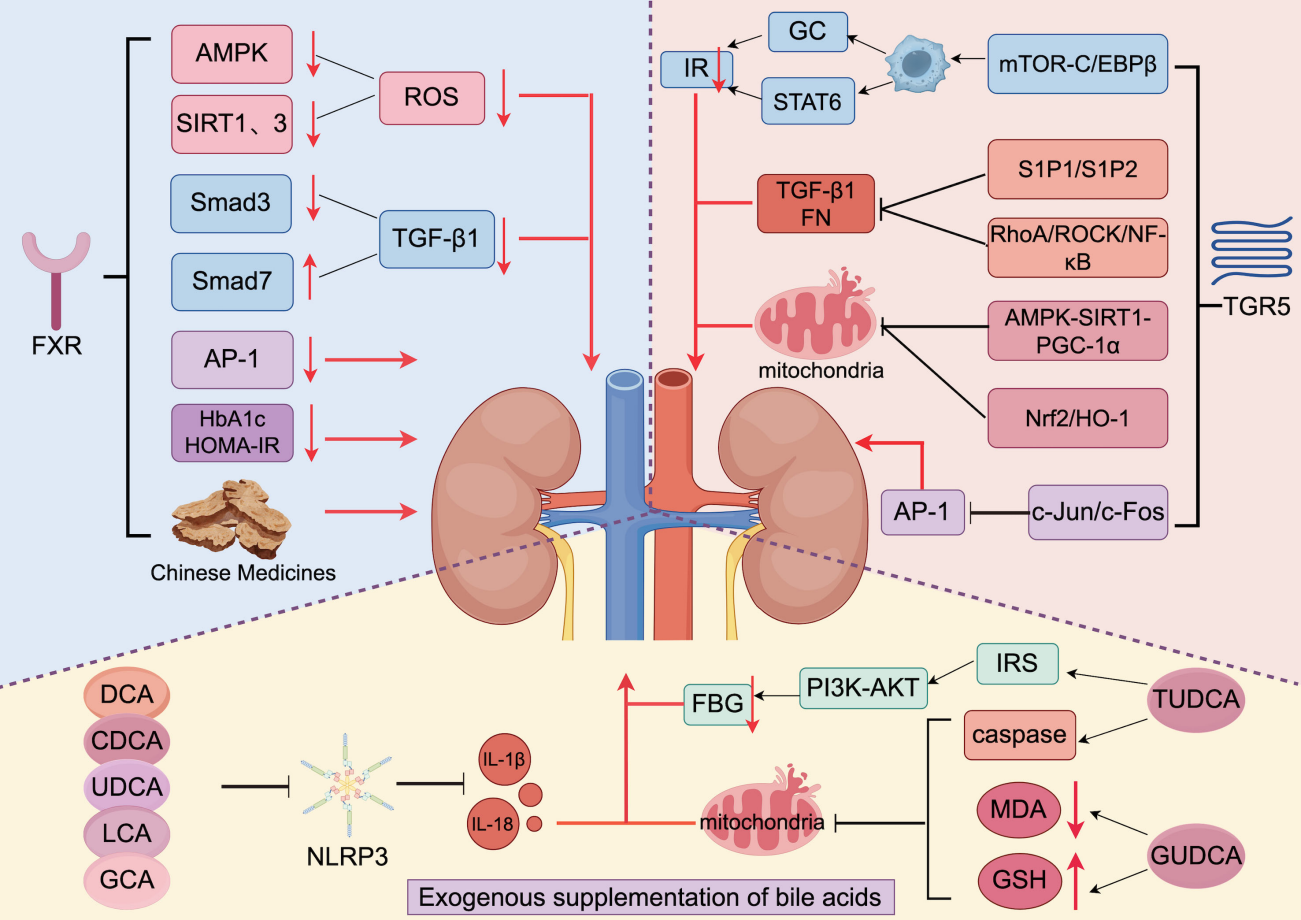
Treatment of DKD via bile acids and traditional Chinese medicines (generated by Figdraw 2.0). Exogenous supplementation with bile acids ameliorates renal inflammation in DKD patients by inhibiting NLRP3, MDA, and GSH and activating IRS. TGR5 is activated to promote the production of GC and STAT6 and reduce the production of TGF-1 and FN by regulating various pathways. Additionally, activated TGR5 inhibits the renal damage that is caused by oxidative stress. FXR is activated to reduce ROS production by inhibiting AMPK and SIRT1. When FXR is activated, it reduces ROS production by inhibiting AMPK, SIRT1, and SIRT3 expression; reduces TGF-1 production by inhibiting Smad3 and promoting Smad7 expression to slow the process of renal fibrosis; and inhibits the expression of AP-1, HbA1c, and HOMA-IR to alleviate renal injury. In addition, traditional Chinese medicine also has a protective effect on the kidneys by interacting with FXR.

Bile acid is both a pathogenic factor and a therapeutic agent. Modulation and regulation of bile acid disorders have significant effect on the treatment of DKD. On the one hand, bile acid can be supplemented externally, and on the other hand, energy metabolism disorders and immune inflammatory responses can be improved by regulating the bile acid receptor ratio (FXR/TGR5); this regulation can alleviate kidney injury and delay the progression of DKD (**Figure 3**).

#### 4.2.1 Exogenous supplementation of bile acids can delay the progression of DKD

Bile acid, a new therapeutic agent, has been shown to play an important regulatory role in glucose metabolism and the inflammatory response (87). Jiang et al. (35) treated DKD mice with cholic acid for 16 weeks and observed significant reduction of proteinuria and glomerulosclerosis by immunofluorescence microscopy. Researchers have shown improvements in inflammation in mice after UDCA supplementation, possibly due to the significant antibacterial effect of UDCA via the TGR5-NF-κB pathway (88). Da Silva et al. (89) reported that TUDCA can improve glucose levels through the activation of IRS-1/PI3K-AKT signaling by binding to IRS insulin binding sites 1 and 2. During an 8-week clinical trial, the researchers found that after oral administration of propionate, secondary bile acid (DCA) levels were elevated, which in turn led to lower cholesterol levels (90). In DKD patients, increased malondialdehyde (MDA) levels and decreased glutathione (GSH) levels are closely related to renal tissue damage. Chen et al. (91) reported that supplementation of GUDCA in patients with glucose metabolism disorder can reduce MDA level and increase GSH level to improve oxidative stress injury and improve the glomerular filtration rate of patients. Protects kidney function. In addition, Guo et al. (92) studied DCA, CDCA, and GCA and the interaction between LCA and macrophages; they found that these bile acids can inhibit the secretion of IL-1β by macrophages through TGR5, reduce the damage to the kidney, and reduce the urine protein content and blood creatinine of patients. The researchers used a bile acid chelator (Sevelamer) in a mouse model of NASH and found that it improves liver fibrosis by regulating gut flora and bile acids. Therefore, we believe that Sevelamer may have a similar effect in reducing kidney inflammation and fibrosis (93). These results indicate that bile acid can be used as a potential therapeutic target for DKD; BA alleviates the inflammatory response, metabolic disorders, and renal tissue injury and dysfunction in DKD patients.

#### 4.2.2 Regulation of bile acid receptors can delay the progression of DKD

##### 4.2.2.1 Regulating FXR delays the progression of DKD

In recent years, FXR is related to regulating the ecological disturbance of intestinal flora, protecting intestinal barrier and improving intestinal antibacterial ability, and is an important way to treat DKD. Gadaleta et al. (94) found that the use of FXR agonists prevented colitius-induced increases in Caco-2 cell permeability and significantly reduced the expression of IL-1β, IL-6, and MCP-1. Xu et al. (95) found that supplementation with Muribaculum and Parasturtella can enhance gut FXR expression and then inhibit cholesterol 7α-hydroxylase (CYP7A1) and sterol 12ahydroxylase (CYP8B1) gene expression in the gut by activating FGF15 via the FXR-SHP axis, thereby promoting bile acid synthesis and reducing cholesterol accumulation. Chen et al. (96) also found that B. fragilis inhibits NLRP3 expression and restores BSH enzymatic activity through FXR, thereby improving intestinal and kidney damage caused by intestinal flora disturbance and abnormal bile acid metabolism.

FXR has received increasing attention for its ability to regulate glycolipid metabolism and alleviate oxidative stress and renal fibrosis (92). In a randomized placebo-controlled trial, the researchers found that supplementation with aronia juice activates FXR and TGR5 through secondary bile acid signaling molecules, which together promote glucose and lipid metabolism (99). After application of the FXR agonist GW4064 to db/db mice for 3 months, the levels of HbA1c and HOMA-IR were reduced, and an insulin resistance test confirmed that FXR ameliorated insulin resistance (100). Xu et al. (98) found that the intestinal FXR antagonist effectively inhibited the expression of PGC-1α in the liver and gluconeogenesis and reduced blood glucose levels. A recent study revealed that vertical sleeve gastrectomy (VSG) altered the gut microbiome distribution and the hydrophobicity of bile acid pools in mice, thereby promoting bile acid metabolism and reducing inflammation and glucose metabolism, while FXR knockout weakened the glycemic improvement induced by VSG (101).

In terms of anti-inflammatory effects, Wang et al. (52) reported that an FXR/TGR5 double agonist (INT-767) increased the activity of AMPK, SIRT1 and SIRT3, all of which play important regulatory roles in reducing ROS production and oxidative stress. This finding provides strong evidence that FXR activation helps maintain mitochondrial homeostasis. Obeticholic acid, a typical FXR agonist, was shown to improve histological features and alleviate fibrosis progression in patients with NASH in a multicenter, randomized, placebo-controlled Phase 3 trial (102). Subsequent studies have shown that activation of FXR also plays a good role in the process of renal fibrosis. Renal fibrosis is an important pathological feature in the progression of DKD to ESRD, and TGF-β1 is an important transforming factor in the process of renal fibrosis. Abnormal expression of TGF-β1 can induce renal fibrosis through the classical Smad pathway, in which Smad3 increases fibrin transcription and promotes tissue fibrosis, while Smad7 inhibits tissue fibrosis. Studies have shown that activation of FXR can inhibit the activation of TGF-β1 pathway by down-regulating the expression of Smad3 and up-regulating the expression of Smad7, thereby alleviating renal fibrosis. FXR can also inhibit the target of AP-1 and antagonize its expression, thereby improving renal fibrosis, thereby increasing glomerular filtration rate, and improving renal injury in DKD patients.

##### 4.2.2.2 Regulation of TGR5 delays DKD progression

Many studies have shown that in both diabetic patients and mouse models, the expression of TGR5 in the kidney is significantly reduced (97) and is inhibited to varying degrees, resulting in serious disorders of blood glucose regulation and inflammatory responses (103). Recently, the researchers reported that they found that the lead compound (77A) of the TGR5 agonist promotes GLP-1 expression, promotes insulin secretion, and lowers blood sugar *in vitro* and in mice (104). After applying TGR5 agonists to diabetic mice, researchers observed that both fasting blood glucose and HbA1c levels were significantly reduced (105, 106). The mechanism may involve the activation of TGR5 by LCA to promote GLP-1 activity and increase glucose uptake by tissues and insulin secretion to stabilize glucose homeostasis. Similarly, increased cholic 7-sulfate was observed in mice during sleeve gastrectomy (SG), and activation of TGR5 induced GLP-1 secretion (107).

Perino et al. (108) reported that TGR5 reduced M1 macrophage chemotaxis by activating mTOR-C/EBP-β but did not affect M2 macrophage polarization. Caratti et al. (109) reported that the GC receptor and STAT6 in M2 macrophages can synergically mediate insulin homeostasis in adipose tissue. Therefore, we hypothesize that TGR5 can regulate the polarization of macrophages, thereby affecting the expression of the GC receptor, preventing severe kidney injury caused by insulin resistance, and delaying the progressive development of DKD. Wang et al. (39)found that eGFR decreased by more than 0.6 ml/min/month in patients with reduced TGR5 expression, and glomerular podocin markers such as Nephrin and Podocin were also reduced, which could be reversed after supplementation with TGR5. Subsequently, Lin et al. (110) found that Smurf1 overexpression could be inhibited by TGR5 supplementation, thereby reducing UTP, Cr, BUN, and NAG activities in DKD patients.

Fibronectin (FN) is known to be an important factor in chemotactic fibroblasts, and its overexpression can cause glomerular basement membrane thickening and accelerate renal tubule fibrosis. Previous studies have shown that NF-κB signaling is mediated by RhoA/ROCK signaling and that TGR5 activation can inhibit RhoA/ROCK/NF-κB signaling in mouse mesangial cells and reduce the expression of FN and TGF-β1, thus alleviating renal fibrosis in DKD mice (111, 112).

### 4.3 Traditional Chinese medicines can regulate the intestinal microbiota-bile acid axis and delay the progression of DKD

At present, the application of traditional Chinese medicine in the treatment of DKD is becoming increasingly extensive, which shows the unique advantages of Chinese medicine in alleviating kidney tissue injury, regulating glucose and lipid metabolism, reducing urinary protein levels, and suppressing the inflammatory response. DKD has no exact term in Chinese medicine but can be classified as “ Oedema and Urinary block and vomiting” according to its pathogenesis and clinical manifestations. The disease occurs in “ Wasting-thirst”, long time dry heat injury of thin fluids, Qi and Yin deficiency pattern, and eventually lead to Qi and Blood and Yin and Yang deficiency pattern. The etiology and pathogenesis of DKD is deficiency of the spleen and kidney pattern as the root, phlegm turbidity blood stasis as the tip. Treatment should pay attention to both the tip and root, kidney and spleen based, with transformation stasis and drain water retention, can effectively reduce the patient’s blood creatinine and urine protein.

Research shows that many TCM monomers could improve blood sugar, lipid levels and early renal function through the “gut-liver-kidney axis”, and reduce pathological damage to kidney tissue. Modern studies have shown that traditional Chinese medicine plays a prominent role in regulating the ecological imbalance of intestinal flora. Shen et al. (113) found that Astragalus membranaceus and Salvia miltiorrhiza increased the abundance of Akkermansia, Akkermansia_muciniphila, Lactobacillus and Lactobacillus muris, among which Akkermansia and Akkermansia_muciniphila were positively correlated with the production of arachidonic acid metabolites. Lactobacillus and Lactobacillus muris are negatively correlated with glycerophospholipid metabolism, which indicates that Chinese medicine can play a positive anti-inflammatory and lipid-lowering role by improving intestinal flora distribution. Liu et al. (114) found that Zicuiyin decoction increased Prevotellaceae and Lactobacillaceae and decreased Enterobacteriaceae, Clostridium and micrococcaceae.

Astragaloside IV (AS-IV), the active ingredient in astragaloside, promotes the release of Nrf2 from Keap1-Nrf2 and reverses the mitochondrial dysfunction induced by high sugar concentrations, thus alleviating podocyte damage caused by oxidative stress (115). In a recent 24-week double-blind randomized trial, researchers found that curcumin can reduce the ratio of firmicute to Bacteroides, regulate intestinal flora homeostasis, increase serum CDCA levels, and thus stimulate the expression of TGR5 in peripheral blood mononuclear cells, playing its therapeutic role (116). Two triterpenoids (alisol M 23-acetate and alisol A 23-acetate) in Alisol can promote the binding of FXR and SHP to play an excitatory role (117). The clinical effect of TCM compounds in the treatment of DKD has been widely verified. Studies have shown that Yinchenhao decoction can improve the abnormal elevation of TCDCA, GCDCA, Tα-MCA and Tβ-MCA and persistent inflammation in mice by regulating FXR (118). Huanglian Jiedu decoction can enhance blood glucose metabolism in DKD rats by regulating the AGE/RAGE/Akt/Nrf2 pathway while reducing triglyceride and low-density lipoprotein cholesterol levels to protect kidney function (119). Danggui Buxue Decoction (120), Danggui Shaoyao SAN (121), Chaihuang Yishen Granules (122) and Yishentongluo have all been shown to inhibit TGF-β1 production and delay renal fibrosis in DKD patients.

## 5 Conclusions

In recent years, an increasing number of researchers have studied the relationships among the intestinal flora, bile acids, and kidney disease, especially the related mechanisms that underlie the progression of DKD. Intestinal flora and bile acids, as novel ways to treat DKD in the future, have great prospects in influencing host epigenetic modification. Bile acid is an important link between the intestinal flora and DKD and regulates energy metabolism and immune homeostasis in the host. Although studies have shown that supplementation of prebiotics and probiotics are effective in the treatment of DKD, for critically ill patients, probiotics, although not harmful, are ineffective in alleviating symptoms and improving prognosis. Although FMT has shown a superior improvement in treatment, many safety factors have not been more accurately studied, such as the risk of donor-transmitted disease and acceptability of the recipient. Many studies have shown that disordered intestinal flora in DKD patients lead to changes in the bile acid pool, metabolic disorders and abnormal expression of bile acid receptors; these phenomena subsequently affect host glucose metabolism and the immunoinflammatory response via different signaling pathways, thereby exacerbating kidney injury in DKD patients. Previous studies have shown that alleviating disordered bile acid metabolism has important potential as a strategy for the treatment of DKD; however, bile acid components are complex and diverse, and additional animal experiments and clinical studies are needed for verification. Therefore, an increasing number of studies have focused on bile acid receptors, and many exciting results have been obtained. FXR/TGR5 activation not only is a clear and highly safe target but also effectively regulates glucose and lipid metabolism, modulates the inflammatory response, and regulates immune homeostasis to delay the progression of DKD. In addition, the clinical efficacy of traditional Chinese medicine has been verified over thousands of years, and modern research has confirmed that these medicines can stimulate FXR/TGR5 and protect the kidney by exerting synergistic effects on multiple targets and pathways. However, given the differences between animals and humans, the design of FXR/TGR5 receptors for use in the clinic is still risky, and more clinical studies are needed to develop safer bile acid receptors in the future; these receptors will provide potential novel directions for the treatment of DKD.
